# Neuroinflammation strokes the brain: A double-edged sword in ischemic stroke

**DOI:** 10.4103/NRR.NRR-D-24-01456

**Published:** 2025-05-06

**Authors:** Giorgia Lombardozzi, Vanessa Castelli, Chiara Giorgi, Annamaria Cimini, Michele d’Angelo

**Affiliations:** Department of Life, Health and Environmental Sciences, University of L’Aquila, L’Aquila, Italy

**Keywords:** brain repair, euinflammation, inflammation, ischemia, mechanisms, microglia, oxidative stress, stroke, therapeutic approaches

## Abstract

Stroke is a major cause of death and disability worldwide. It is characterized by a highly interconnected and multiphasic neuropathological cascade of events, in which an intense and protracted inflammatory response plays a crucial role in worsening brain injury. Neuroinflammation, a key player in the pathophysiology of stroke, has a dual role. In the acute phase of stroke, neuroinflammation exacerbates brain injury, contributing to neuronal damage and blood–brain barrier disruption. This aspect of neuroinflammation is associated with poor neurological outcomes. Conversely, in the recovery phase following stroke, neuroinflammation facilitates brain repair processes, including neurogenesis, angiogenesis, and synaptic plasticity. The transition of neuroinflammation from a harmful to a reparative role is not well understood. Therefore, this review seeks to explore the mechanisms underlying this transition, with the goal of informing the development of therapeutic interventions that are both time- and context-specific. This review aims to elucidate the complex and dual role of neuroinflammation in stroke, highlighting the main actors, biomarkers of the disease, and potential therapeutic approaches.

## Introduction

Stroke is the most common and acute manifestation of cerebrovascular disease, characterized by the occlusion of blood vessels. It is the second leading cause of death and the third most common cause of disability worldwide (Rajsic et al., 2019; Kuriakose and Xiao, 2020; Feigin et al., 2022). Stroke can be classified into two main categories: ischemic stroke, caused by the disruption of blood supply in the brain, which accounts for 80% of cases, and hemorrhagic stroke attributed to the rupture of a blood vessel or a vascular structure, constituting 20% of cases (Donkor, 2018). Between 1990 and 2016, the incidence of stroke experienced a sharp increase in low- and middle-income countries, where it doubled. In contrast, high-income countries saw a significant decline. According to the Global Burden of Disease Study (GBD), although the prevalence of stroke has decreased, factors such as the age of affected individuals, their gender, and geographic location have contributed to an increase in the socioeconomic burden of stroke over time (GBD 2016 Stroke Collaborators, 2019; Kleindorfer et al., 2021). The prevention of stroke requires modifying risk factors both at the individual and community levels, while the treatment of stroke is based on managing its pathophysiology. Currently, there are a few treatment options available, among which the use of tissue plasminogen activator is most effective when administered within 4.5 hours of the ischemic event. Additionally, some side effects of tissue plasminogen activator are known, including an increased risk of cerebral hemorrhage (Knecht et al., 2017). Regarding the pathophysiology of ischemic stroke, it arises from the occlusion of one of the major cerebral arteries or their collateral branches, leading to diminished blood flow in a specific region of the brain. Reduced blood flow leads to cerebral infarction in the area supplied by the affected vessel (Catanese et al., 2017). The characteristic pathophysiological changes of ischemic stroke begin with a sudden interruption of blood flow, leading to a rapid onset of neurological deficits. Clinical symptoms typically appear within 24–48 hours and are contingent upon the affected area and the fate of the surrounding ischemic zone, known as the penumbra (Feng et al., 2019). Oxygen and nutrient deficiencies trigger a complex series of biochemical and molecular events, which eventually lead to widespread ischemic cell death in the affected brain tissue (Simats and Liesz, 2022).

In cerebral ischemia, there are many responses and activated pathways, including inflammation, oxidative stress, and excitotoxicity. Inflammation is a crucial factor in all stages of stroke development, from vessel occlusion in the acute phase to post-ischemic repair (Mo et al., 2020). Neuroinflammation can cause additional damage leading to cell death, however, it also exerts a beneficial role in stimulating remedial action (Maida et al., 2020a). Immune mediators are the origin of signals with a pro-inflammatory position that can stimulate the cells in the brain leading to the penetration of numerous inflammatory cytotypes (different subtypes of T-cells, neutrophils, monocytes/macrophages, and many inflammatory cells) within the area affected by ischemia. This process is responsible for further ischemic damage to the brain and the severity and extent of cerebral neuroinflammation vary depending on the area, depth, intensity, and progression of the ischemic lesion (Pluta et al., 2021). Regarding its positive role, upon ischemia, a strong and profound neuroinflammatory response is initiated to remove the injured tissue and prepare the brain for repair. Neuroinflammatory responses in ischemic stroke are also involved in the recovery of neuroplasticity and motor dysfunctions (Candelario-Jalil et al., 2022). More research is necessary to mechanistically comprehend when the neuroinflammatory responses begin the transition from injury to repair. Understanding the underlying mechanisms and timing of neuroinflammation is crucial for developing effective treatments for this disorder. This review aims to discuss the dual role of inflammation in the progression of ischemic damage, highlighting the main actors, biomarkers of the disease, and potential therapeutic approaches.

## Search Strategy

For this work, the primary bibliographic citation databases used were PubMed and Google Scholar. The searches were conducted on these platforms between September and November 2024, to ensure inclusion of the most recent and relevant literature. Search limits were applied to focus on peer-reviewed articles, specifically original research studies. Keywords were used to refine the results, targeting terms including “neuroinflammation,” “ischemic stroke,” “microglia,” and “brain repair.”

## Neuroinflammation in Ischemic Stroke

Neuroinflammation is the process through which the brain’s innate immune system is activated in response to an inflammatory stimulus, such as that caused by cerebral ischemia (Jurcau and Simion, 2021). In the brain, this inflammatory response, called neuroinflammation, is a fundamental reaction generated to protect the central nervous system; however, uncontrolled, or prolonged neuroinflammation is potentially harmful and can cause cellular damage (Drieu et al., 2018; Jurcau and Simion, 2021). This process originates in microcirculation and involves a diverse range of cell types, including innate immune cells such as microglia and adaptive immune cells such as lymphocytes (Iadecola et al., 2020; Maida et al., 2020a). Additionally, it involves activating molecular mediators. The timeline of inflammatory events occurring during cerebral ischemia can be summarized into three main phases. In the first hours following the onset of the ischemic event, there is an acute phase characterized by tissue damage resulting from the blockage of blood flow, leading to deprivation of oxygen and nutrients known to be essential for neuronal survival (Drieu et al., 2018; Jurcau and Simion, 2021; DeLong et al., 2022). Following cerebral ischemia, disruption of the integrity of the blood–brain barrier (BBB) and loss of tight junctions between endothelial cells are observed (Rayasam et al., 2018). Assessing blood–brain barrier disruption can aid in the early stratification of hemorrhagic risk in patients with ischemic stroke, especially those treated with intravenous thrombolysis and/or endovascular procedures (Arba et al., 2021). Breakdown of the blood barrier and loss of joints facilitates leukocyte diapedesis in damaged brain tissue (Rayasam et al., 2018). Leukocytes that migrate in the cerebral parenchyma can cause brain damage in different ways, such as by producing reactive oxygen species, proteases, and matrix metalloproteases (Pluta et al., 2021). During this phase, microglia are activated, which is a cell population that actively participates in the inflammatory process during the ischemic stroke (Drieu et al., 2018). During the initial few days following an ischemic stroke, there is a phase known as subacute that marks the resolution of inflammation. The final stage is the late phase (days to weeks after the stroke), which is designed to restore the tissue’s integrity and support the formation of the glial scar. In this process, astrocytes are involved, ensuring the protection of pre-existing blood vessels, and promoting repair and vascular remodeling. The glial scar acts as a barrier between the site of the injury and healthy tissue, preventing the harmful factors produced by the damaged area from spreading to other areas (Williamson et al., 2021; Cao et al., 2023).

Neuroinflammatory changes in the brain are evident across every stage of an ischemic event, starting from the initial halt in cerebral blood flow to the advanced phases of tissue recirculation in the affected brain areas (Radenovic et al., 2020).

### Main actors in ischemic stroke: impact of inflammatory cells

In the development of ischemic stroke, a multitude of cells is engaged, actively contributing to the inflammatory response. The interest in these cells stems from the dual role that most of them play in the inflammatory process. The microglial response is one of the earliest immune responses to activate following a stroke. Microglial cells are mononuclear phagocytes residing in the brain parenchyma, constituting 10%–15% of immune cells (Drieu et al., 2018; Xu et al., 2020; Gao et al., 2023). The microglia thus play a crucial role in maintaining homeostasis in a healthy brain by clearing away unwanted synapses. In response to ischemic stress, they are rapidly activated: they recognize extracellular ATP released by damaged cells as a “find-me” signal and phosphatidylserine exposed on the membranes of damaged cells as an “eat-me” signal (Neher et al., 2013; Nakamura and Shichita, 2019). Microglia can be characterized as small cells with broad protruding branches when inactive. However, following brain ischemia, microglia are activated, resulting in observable changes in their phenotypes (Guruswamy and ElAli, 2017; Maida et al., 2020b). Specifically, they assume four distinct morphological states that indicate an increase in activation: a branched state, when they are in the quiescent phase with a cellular body and long processes; an intermediate state, when they are active with a magnified cell body and shorter processes; ameboid state, with very short or absent processes and finally, a round state, morphologically similar to macrophages, the most active microglial representative form (Anttila et al., 2017). The activation of microglial cells occurs at a peak between 2–3 days and lasts for several weeks following the stroke onset. To better understand the role of microglia in ischemic stroke, it is essential to consider their intrinsic heterogeneity and plasticity. In particular, microglial cells can simultaneously express varying levels of pro-inflammatory and anti-inflammatory markers, highlighting their ability to exhibit a spectrum of phenotypic subtypes rather than being strictly confined to two distinct states, as previously classified (Masuda et al., 2020; Ochocka and Kaminska, 2021). This capacity stems from their ability to adopt a wide range of activation states and phenotypes in response to different stimuli and microenvironmental signals, which significantly influence their functions and their impact on neuronal survival and recovery (Haupt et al., 2024).

Microglia play a dual and context-dependent role in ischemic stroke. In aging, they adopt a dysfunctional phenotype marked by impaired phagocytosis, reduced motility, and heightened inflammatory responses to injury. This exaggerated inflammatory response after ischemic stroke is accompanied by increased production of reactive oxygen species, as observed in aged mice. While studies show that depleting microglia can reduce neuroinflammation in conditions such as intracerebral hemorrhage and Alzheimer’s disease, similar interventions in ischemic stroke models have yielded adverse outcomes (Sosna et al., 2018; Lee et al., 2020). Specifically, microglial depletion in aged male mice led to worsened brain damage, larger infarcts, and heightened peripheral immune cell infiltration. These findings underscore the complex and critical role of microglia in mediating inflammation and recovery in ischemic stroke (Lee et al., 2020).

Another type of cell involved in stroke is astrocytes. Astrocytes are a subset of the non-neuronal glial cells group, which includes oligodendrocytes, Schwann cells, and microglia. Astrocytes perform specific functions in safeguarding and maintaining neuronal cells through the regulation of ion balance, oxidative stress, clearance of neurotransmitters, waste, and metabolic by-products (Bylicky et al., 2018; Shen et al., 2021; Williamson et al., 2021). The brain damage resulting from ischemic stroke affects the absorption of glutamate by astrocytes, which under normal conditions is converted into glutamine for neuronal reuse (Bylicky et al., 2018; Jayaraj et al., 2019). Astrocytes, in addition to providing neurotrophic support, are structurally connected to the endothelial cells of brain capillaries and pericytes that make up the BBB. During ischemic events, matrix metalloproteinase 9 (MMP-9) interrupts this connection, leading to BBB breakdown and subsequent invasion of peripheral inflammatory cells (Manu et al., 2023).

The interplay between astrocytes and microglia plays a pivotal role in modulating central nervous system inflammation during ischemic stroke. This bidirectional communication occurs through the release of various cytokines and inflammatory mediators. This interaction is critical in determining the balance between detrimental and reparative processes, emphasizing their potential as targets for therapeutic strategies in ischemic stroke (Linnerbauer et al., 2020; Tschoe et al., 2020).

The brain parenchyma is first populated by blood immune cells, such as leukocytes and neutrophils, which peak 48–72 hours after an ischemic event. Leukocytes are responsible for releasing proinflammatory factors in the ischemic area of the brain, and leukocytosis can be a sign of the post-ischemic stroke inflammatory response (Jayaraj et al., 2019). The different types of leukocytes contribute to brain damage in various ways. Leukocytes adhere to the endothelial wall, causing the so-called plugging in erythrocyte flow responsible for the no-reflow phenomenon. Additionally, leukocytes produce proteases, reactive oxygen species, and matrix metalloproteinases that can significantly damage blood vessels and brain tissues. Biologically active substances such as eicosanoids, leukotrienes, and prostaglandins, which are produced when leukocytes are activated by phospholipases, induce vasoconstriction and platelet aggregation (Jurcau and Simion, 2021; Pluta et al., 2021). Colonization by neutrophils of brain tissues begins within six hours of the onset of ischemia and may persist for up to 6 days. Segel et al. (2011) have shown that the extent of infiltration is associated with the severity of neurological damage.

Although the majority of studies identify pathological roles played by neutrophils, some studies suggest positive effects and benefits following stroke (Segel et al., 2011). The depletion of neutrophils decreases the levels of various growth factors, including brain-derived neurotrophic factor and vascular endothelial growth factor, attenuating focal angiogenesis induced in the mature mouse brain (Hao et al., 2007). Neutrophils are a significant source of MMP-9 within the first 24 hours of ischemic stroke. MMP-9 levels increase in plasma within the initial 2–6 hours post-stroke, leading to BBB disruption (Jickling et al., 2015; Chen et al., 2021). Neutrophils also play a reparative role during stroke, through the release of anti-inflammatory molecules and their removal. A specific subgroup of neutrophils, known as N2 neutrophils, exhibit anti-inflammatory properties. The shift from the pro-inflammatory phenotype of N1 neutrophils to the anti-inflammatory phenotype of N2 occurs when neutrophil levels reach high levels in the central nervous system (Cuartero et al., 2013; Jickling et al., 2015).

These pro- and anti-inflammatory agents act as predictors of ischemic damage. However, other biomarkers, such as cytokines, also contribute to brain damage and repair, but the balance between the beneficial and harmful effects of cytokines largely depends on the biochemical and physiological state of the brain.

### Investigating inflammatory biomarkers in ischemic stroke

Among the biomarkers of inflammation, we can observe cytokines, small polypeptides that are normally expressed at low levels, which mediate the inflammatory response or can act as anti-inflammatory molecules. After the ischemic event, there is an overexpression of cytokines in the brain, with their production coming from both immune system cells and resident brain cells, such as neurons and microglia (Ramiro et al., 2018). Cytokines play a significant role in the progression of inflammation, with both pro-inflammatory and anti-inflammatory properties (Jurcau and Simion, 2021; Liberale et al., 2021). The three main pro-inflammatory cytokines include interleukin 1β (IL-1β), IL-6, and tumor necrosis factor alpha (TNF-α). TNF-α is among the first cytokines to appear during the inflammatory response following cerebral ischemic injury; therefore, it is useful in defining the onset of the inflammatory reaction and in evaluating the resolution of the damage. Its expression levels are increased both in cerebrospinal fluid and serum (Zaremba and Losy, 2001; Maida et al., 2020a; Kowalski et al., 2023). It presents an initial peak within the first hours (1–3 hours), followed by a subsequent peak after more than 24–36 hours (Maida et al., 2020a). Within 6 hours of stroke onset, there is an increase in serum levels of TNF-α, which remain elevated for 10 days (Zaremba and Losy, 2001).

IL-1β is a pro-inflammatory cytokine with neurotoxic effects, which can interact with vascular endothelium, increasing leukocyte adhesion and promoting edema formation (Maida et al., 2020a). The levels of IL-1β and TNF-α are found to increase in the cerebrospinal fluid, peaking in the days following the stroke. As for its expression in serum and plasma, no trace has been detected, likely due to its localization at the inflammatory site (Sobowale et al., 2016).

IL-6, one of the pro-inflammatory proteins, plays multiple roles in the brain parenchyma, with effects that can be either harmful or beneficial. Various other molecules, such as prostaglandins, TNF-α, IL-1, and IL-4, can influence its secretion (Maida et al., 2020a; Bitencourt et al., 2022).

Among the anti-inflammatory cytokines, we can distinguish IL-10, which is produced by various types of cells, including monocytes, CD4 cells, CD25 cells, regulatory T cells, and mast cells. During ischemic stroke, an increase in IL-10 expression is observed, with a peak occurring 3 days after the onset of the stroke (Jurcau and Simion, 2021; Gao et al., 2023). IL-10 plays a critical role in containing brain damage during cerebral ischemia; in fact, it can suppress the excessive secretion of pro-inflammatory cytokines (Maida et al., 2020b; Piepke et al., 2021).

The transforming growth factor-beta (TGF-β) is a growth factor widely distributed in the human body. In particular, the TGF-β3 isoform plays a crucial role in supporting neuronal survival and promoting brain tissue restoration. It should inhibit the function of both neutrophils and astrocytes, thereby indirectly reducing the production of pro-inflammatory cytokines. Additionally, it can inhibit microglia, thus helping to reduce the potential damage associated with it (Maida et al., 2020b).

Chemokines are small molecules (8–10 kDa) belonging to the cytokine family. Specifically, cytokines such as IL-1β and TNF-α induce the production and release of specific chemokines, such as CINC (cytokine-induced neutrophil chemoattractant, a neutrophil attractor), MCP-1 (monocyte chemoattractant protein-1, a monocyte attractor), fractalkine, MRF-1 (microglial response factor-1), and MIP-1 (macrophage inflammatory protein-1), whose levels are overexpressed in the early hours following the ischemic event and persist for up to 6 hours after onset. Their distinctive feature is the ability to attract leukocytes through chemotactic activity during the inflammatory response (Ceulemans et al., 2010; Jurcau and Simion, 2021). To achieve leukocyte recruitment, chemokines cooperate with adhesion molecules and influence the permeability of the BBB to ensure diapedesis through the vessel wall (Ceulemans et al., 2010).

## Dual Nature of Neuroinflammation: Balance Between Protection and Harm in Ischemic Stroke

In the context of cerebral ischemia, neuroinflammation emerges as a complex process, playing an ambivalent role characterized by both positive and negative impacts on the affected brain tissue. Brain inflammation following ischemic events was traditionally considered harmful, new evidence suggests that some aspects of the inflammatory response may contribute to brain protection.

### Double role of cytokines and inflammatory molecules: pro-inflammatory and protective mechanisms

In the ischemic brain, there is a predominant increase in the production of cytokines, reactive oxygen species, and other inflammatory mediators such as inducible nitric oxide synthase, resulting in the recruitment of peripheral macrophages and neutrophils to the site of injury (DiSabato et al., 2016).

In the physiological state, there is a delicate balance between pro-inflammatory and anti-inflammatory factors, which is drastically disrupted during the early stage of acute ischemic stroke. Inflammation represents a fundamental event not only in the onset but also in the progression of ischemic damage. From a study conducted by Basic Kes *et al.* (2008), an association between levels of pro and anti-inflammatory cytokines and the inflammatory response in acute ischemic stroke has emerged. Furthermore, their relationship with the severity and hence the outcome of the pathology has been reported (Basic Kes et al., 2008). Specifically, it has been indicated that stroke patients had significantly higher levels of pro-inflammatory IL-6 compared to controls, 12 hours after stroke onset. Conversely, the levels of the anti-inflammatory IL-10 were found to be reduced in the initial phase of ischemic stroke, leading to a worse clinical outcome (Basic Kes et al., 2008; Maida et al., 2020a). Indeed, IL-10 plays a relevant role in ischemic stroke, suppressing pro-inflammatory signals and dampening immune responses. From the study conducted by Pérez-de Puig et al. (2013), the beneficial role of IL-10 following stroke was highlighted in transgenic mice overexpressing this IL, exhibiting smaller infarct areas. The main cells involved in the ischemic event, as previously discussed, are characterized by their wide heterogeneity and numerous functions within the context of inflammation. TNF-α exerts a dual effect on brain inflammation following the initial stages of ischemia, acting as an inflammatory promoter in the early phases and as an immunosuppressive agent in the chronic phase of ischemic stroke (Zaremba and Losy, 2001).

### Discovering the dual impact of microglia and astrocytes

Following an ischemic event, microglial cells rapidly activate and serve as the brain’s primary immune effectors, responsible for immune surveillance and neuroprotection (DiSabato et al., 2016; Wang et al., 2022). Recent *in vivo* experiments have provided further insights into the complex involvement of microglia in the pathobiology of ischemic stroke. According to a study, implantation of microglia in culture in ischemic brain tissue led to a reduction in damage and improved functional performance (Kitamura et al., 2004). Additionally, microglia not only limit the expansion of the lesion but also promote tissue repair and regeneration after the ischemic event. It has been observed that microglia in the brain regions involved in adult post-stroke neurogenesis, specifically in the subventricular zone, are active and proliferating for several weeks (Weinstein et al., 2010; Guruswamy and ElAli, 2017).

A study conducted on the treatment with an inhibitor of colony-stimulating factor 1 receptor, involved in the repopulation of microglial cells, has allowed studying the effects resulting from the absence of microglia. In this study, they showed that the selective removal of microglia causes a substantial increase, amounting to 60%, in the size of the infarct, an event reversed by the subsequent repopulation of these cells. Additionally, it has been observed that microglia limits excitotoxic damage occurring after ischemic stroke, as evidenced by the assessment of the high calcium concentration and the elevated neuronal mortality that follows microglial reduction (Szalay et al., 2016). Microglial cells promote neurogenesis and oligodendrogenesis by reducing the production of TNF-α and increasing the production of insulin-like growth factor 1 (IGF-1) (Butovsky et al., 2006; Sierra et al., 2014).

The activation of microglia occurring in the ischemic core following the lesion is triggered by excitotoxic signals generated during the ischemic cascade, while in the peri-infarct regions, microglial activation is triggered by damage-associated molecular patterns (DAMPs), which elicit a strong inflammatory response. Additionally, microglia can be activated through their purinergic receptors that recognize and bind extracellular nucleotides adenosine triphosphate (ATP) and adenosine diphosphate (ADP) released by dysfunctional neurons within the damaged tissue (Anttila et al., 2017).

Once activated, microglia alter their phenotype by adopting various morphologies closely associated with spatial structural damage and by adopting an altered gene expression pattern that induces cellular polarization towards functionally distinct phenotypes, involved in pro-inflammatory and anti-inflammatory actions (Guruswamy and ElAli, 2017).

Astrogliosis is also beneficial as it promotes calcium propagation to enhance signal conduction and ensure reparative and protective action, but it can also lead to an increase in the presence of various neurotoxic substances in case of excessive activation (Pluta et al., 2021). The response of astrocyte cells can represent a detrimental event as the glial scar formed in the early stages of ischemic stroke acts as a barrier that separates the lesion site from the surrounding vital tissue, hindering axonal regrowth and reinnervation (Barreto et al., 2011; Malone et al., 2019).

### Exploring the protective role of immune conditioning in inflammation

A positive aspect of inflammation is represented by “euflammation.” “Euflammation” is a term used to describe a type of immune conditioning where peripheral inflammation is induced without the typical symptomatic characteristics of inflammation, such as pain, swelling, or redness. This phenomenon has been investigated in the context of reducing the negative side effects of inflammation, particularly upon cancer treatments (DiSabato et al., 2016; Bever et al., 2017). Liu et al. (2016) conducted interesting studies on animals pretreated with repeated subthreshold infectious stimulations at the peripheral level (i.e., euflammation). Notably, they reported a reduced expression of cytokines in the brain and lower circulating cytokines (3 hours post-infection), as well as morphological changes in microglia (24 hours post-infection) following intraperitoneal administration of LPS or *Escherichia coli* (Liu et al., 2016). Euflammation demonstrates that prior exposures to a specific non-pathogenic antigen enable the organism to develop long-term memory. This immune preconditioning can determine the expression of a distinctive microglial profile, characterized by increased anti-inflammatory or reparative activity, capable of providing a protective contribution against inflammatory conditions that occur during damage (Tarr et al., 2014).

Specifically, microglial cells can adopt a distinctive profile characterized by reduced production of pro-inflammatory cytokines (e.g., IL-1β and TNF-α) and increased anti-inflammatory mediators such as IL-10. This shift promotes a reparative and neuroprotective environment, mitigating the harmful effects of inflammation in pathological conditions such as ischemic stroke or neurodegenerative diseases (Tarr et al., 2014). This approach could pave the way for targeted interventions aimed at modulating immune responses in chronic inflammatory diseases or contexts of acute injury, supporting the idea that inflammation is not merely a pathological phenomenon but also a crucial adaptive mechanism with significant implications for health and disease.

## Immunomodulatory Approaches to Treating Ischemic Stroke

The main treatment strategies for ischemic stroke focus on regulating the immune system, with specific attention to the involved cells, the inflammatory responses, and the possibility of inhibiting them to counteract the progression of stroke.

### Directing focus toward microglia and astrocytes

The recombinant human fibroblast growth factor 21 (rhFGF21) acts as an endocrine regulator, modulating the inflammatory responses of microglial and macrophage cells involved in acute stroke. In the study conducted by Wang et al. (2020), intraperitoneal administration of rhFGF21 in C57BL/6 mice subjected to middle cerebral artery occlusion (MCAO) induced a significant reduction in infarct size and an improvement in neurological deficit. rhFGF21 resulted in an attenuation of microglial polarization towards the M1 phenotype, which is known to have pro-inflammatory effects, but did not demonstrate any effect on the M2 phenotype (anti-inflammatory phenotype) in the acute phase of the MCAO. Additionally, rhFGF21 decreased certain pro-inflammatory gene expressions (TNF-α, IL-1β, IL-6, and TGF-β).

Among the valid therapeutic options for modulating the inflammatory response, antibiotics are well-known. At the preclinical level, minocycline, along with the free radical scavenger and hyperbaric oxygen, plays a neuroprotective role due to reduced activity of the M1 phenotype (Chen et al., 2014; Zheng et al., 2022). Minocycline, a tetracycline-derived antibiotic, is recognized as a therapeutic strategy targeting microglia, inhibiting its activation and demonstrating efficacy in brain protection against ischemia (Anttila et al., 2017). It reduces inflammation and shields neurons from programmed cell death. Additionally, minocycline can counteract the formation of free radicals and inhibit MMP, thus contributing to long-term brain health (Anttila et al., 2017; Drieu et al., 2018).

The study conducted by Pawletko et al. (2023) demonstrated the beneficial effect resulting from the single administration of the antibiotic minocycline at low dosage in rats with ischemic stroke. Minocycline was able to increase neuronal vitality and to reduce neurodegeneration, leading to a reduction in the volume of the infarct area (Pawletko et al., 2023). Minocycline succeeded in two Phase II human studies, which confirmed the efficacy and safety of the drug (Lampl et al., 2007; Padma Srivastava et al., 2012). Conversely, in another study conducted by Kohler et al. (2013), minocycline did not demonstrate similar efficacy, confirming the need for a deeper understanding of the transient M1/M2 phenomenon involving microglia.

Minocycline is also a potent inhibitor of MMPs, whose increase is known to be correlated with neuronal apoptosis, leukocyte infiltration, BBB disruption, edema, and hemorrhage (Knecht et al., 2017). There are several MMPs implicated in infarction including MMP-2, MMP-3, MMP-7, and MMP-9. The focus on MMP-9 stems from its role in mediating the most intense and irreversible damage to the BBB in ischemic events (Malone et al., 2019). A study has shown that higher levels of MMP-9 in the serum at the onset of stroke could serve as a predictor of unfavorable stroke outcomes (Abdelnaseer et al., 2017).

The astroglial response begins at the infarct site as early as 4 hours after the ischemic event, leading to profound changes in astrocyte morphology, upregulated expression of glial fibrillary acidic protein, and increased intracellular calcium level (Kim et al., 2016; Petrovic-Djergovic et al., 2016).

A previous study has shown that reduced astrogliosis is correlated with a decrease in the size of the infarcted area, while other studies highlighted the detrimental role of these astrocytic cells (Barreto et al., 2011). Transplantation of genetically modified astrocytes represents a valid and innovative therapeutic strategy because it is capable of activating endogenous neuroprotective mechanisms (such as neurogenesis, angiogenesis, and inflammation regulation) to promote recovery of axonal myelination, modulate the immune response, and facilitate the release of neurotrophic factors that prevent oxidative stress and excitotoxic damage (Becerra-Calixto and Cardona-Gómez, 2017).

### Focus on inflammatory responses

Given the role played by immune cells in the inflammatory process affecting the brain following ischemic stroke, it is interesting to evaluate immunomodulatory strategies targeted at them (Xie et al., 2024).

Since TNF-α is an immunomodulatory cytokine involved in neuroinflammation and neuronal damage in response to ischemia, thus the use of TNF-α inhibitors could be beneficial in the treatment of ischemic stroke. Adalimumab, infliximab, and etanercept, among other anti-IL-6 agents, are currently under consideration for the prevention of cardiovascular disease (Kim et al., 2016; Petrovic-Djergovic et al., 2016). Etanercept is a protein compound formed by coupling the TNF receptor with the Fc portion of immunoglobulin G, acting as a TNF inhibitor, thereby reducing the neurotoxic activation of microglia caused by this cytokine (Cao et al., 2023). The combined use of the recombinant human IL-1 receptor antagonist (rhIL-1Ra) and the TNF-α receptor antagonist, etanercept, has shown a significant reduction in infarct size and improvement in neurological outcomes. Even cytokines considered beneficial, such as IL-6 and IL-10, which promote angiogenesis and functional recovery, are potential therapeutic targets for ischemic stroke (Cao et al., 2023). The IL-1 receptor antagonist (IL-1Ra), a selective and natural competitive inhibitor of IL-1, holds promise as an innovative treatment for stroke, as it can reduce infarct volume and improve long-term functional outcomes in experimental stroke models (Pradillo et al., 2017).

Interestingly, recent research has highlighted the protective potential of plant-derived compounds belonging to the flavonoid class against cerebral ischemic damage by modulating inflammatory responses. Flavonoids, abundant in our diet, limit neuroinflammation resulting from ischemic stroke by directly acting on the activation of microglia and/or astrocytes. An example is represented by quercetin, the main representative of the flavanol class, whose administration in rats following ischemic stroke significantly reduced pro-inflammatory cytokines (IL-1β and IL-6) and increased anti-inflammatory ones (IL-4, IL-10, and TGFβ1), demonstrating neuroprotective effects. Similarly, other flavanols such as apigenin or rutin have shown their anti-inflammatory effects, manifested in the reduction of pro-inflammatory factor release (Lu et al., 2024). Rutin can reduce the expression and activity of MMP-9 and promote proper functioning of the BBB in rats with focal cerebral ischemia (Negahdari et al., 2021). Some immunomodulatory strategies aim to regulate inflammatory responses and redox reactions caused by ischemic stroke. The combined mixture of a natural terpene, such as borneol, with an antioxidant drug, such as edaravone, has demonstrated neuroprotective functions against ischemic lesions. This combination can slow down immune cascades induced or associated with neuroinflammation and regulate the inflammatory response, providing additional neuroprotective effects (Xu et al., 2021; Cao et al., 2023).

The 4-ethylguaiacol (4-EG), a phenolic compound belonging to the class of methoxyphenols, suppresses inflammatory immune responses. This compound, found in wine and beer and identified in various foods including peppers, corn, sesame, and coffee, exerts anti-inflammatory effects by inhibiting nuclear factor kappa B (Zhao et al., 2019; Weng et al., 2022). The treatment with 4-EG in vivo studies conducted on MCAO mice reduced the size of cerebral infarction, limited blood-brain barrier lesions, improved neurological dysfunction, and increased survival (Weng et al., 2022).

Estrogens, particularly 17β-estradiol, provide a protective role against brain damage resulting from ischemic stroke. This hormone exerts its effects by acting on both the local and systemic immune systems (Zhong et al., 2023). Specifically, 17β-estradiol can inhibit the production and release of certain pro-inflammatory cytokines, including IL-1β, IL-6, and TNF-α. This property makes it a promising candidate for new therapeutic strategies for ischemic stroke (Ritzel et al., 2013; Zhong et al., 2023).

Acupuncture proves to be a valid alternative for treating ischemic stroke due to its beneficial effects on various aspects of neurological recovery. It can modulate inflammatory signaling pathways, such as TLR and Notch receptors, thereby reducing inflammation, infarct volume, and ischemia/reperfusion damage. Clinical studies further suggest that acupuncture decreases inflammatory cytokines, including IL-1β and C-reactive protein, enhancing neurological rehabilitation. Unlike therapies targeting single mechanisms, acupuncture acts through multiple channels and targets, offering a holistic approach to managing inflammation and restoring neural functions (Cao et al., 2020). The work conducted by Ren et al. (2024) confirmed the therapeutic role of electroacupuncture in the treatment of ischemic stroke, with a particular focus on neuroinflammation. Electroacupuncture significantly reduced neuroinflammation by modulating microglial activation and decreasing the levels of pro-inflammatory cytokines, such as TNF-α and IL-1β. This beneficial effect can be attributed to the inhibition of the transient receptor potential vanilloid 4 channel, which appears to be a key mediator in amplifying inflammation during ischemic stroke (Ren et al., 2024).

Ginsenoside-Rd, derived from ginseng used in traditional Chinese medicine, has shown significant neuroprotective effects in ischemic stroke. This compound can cross the BBB, enhancing its impact on brain tissue (Ratan et al., 2021; Cheng et al., 2023). In experimental stroke models, treatment with ginsenoside-Rd reduced infarct volume preserved neuronal function, and improved neurological outcomes (Zhang et al., 2014; Ratan et al., 2021). Studies, including clinical trials, have confirmed its effectiveness in reducing brain damage by inhibiting inflammation and decreasing microglial activation. Clinical research further validated these findings, showing improvements and highlighting its potential to aid recovery in patients with acute ischemic stroke (Liu et al., 2012; Ratan et al., 2021).

### Therapeutic approaches to target leukocyte infiltration

Specific therapies targeting lymphocytes could hold promising outcomes as they address the late stage of brain injury, offering a potentially larger and more significant therapeutic window. Valid candidates are represented by regulatory T cells, known by the acronym Treg, which are categories of cells that play the role of endogenous regulators in modulating immune responses in the ischemic brain (Zhang et al., 2018; Liu et al., 2023). Regulatory T cells (Tregs) typically represent 5%–10% of the CD4^+^ T cell population and are distributed within lymphoid organs as well as at sites of inflammation (Yamamoto et al., 2022). The protective role of Treg cells after cerebral ischemic injury has been demonstrated in numerous studies (Li et al., 2013; Liesz and Kleinschnitz, 2016).

The intravenous administration of Treg up to 24 hours after the ischemic event resulted in a reduction of the infarction size and an improvement in ischemic pathogenesis characteristics. In particular, the Treg attenuated the interruption of the BBB by suppressing the MMP-9 and thus preventing the infiltration of peripheral inflammatory cells (Li et al., 2013). The number of Treg cells following the ischemic event decreases rapidly and then increases significative in the following weeks (Malone et al., 2019). To limit this feature and implement the clinical use of this cell class, a study has yielded promising results following the administration of the IL-2/IL-2 antibody complex (IL-2/IL-2Ab). IL-2/IL-2Ab has been shown to have protective effects in stroke-induced damage by increasing the number and effectiveness of Tregs (Zhang et al., 2018).

The Treg cells provide early protection to the central nervous system, mitigating the harmful actions of peripheral immune cells rather than directly influencing central nervous system cells (Li et al., 2014). An immunomodulatory drug is represented by Fingolimod, which acts as a modulator of the sphingosine-1 phosphate receptor, preventing the exit of lymphocytes from the lymph nodes (Iadecola et al., 2020). Although Fingolimod is used as therapy in relapsing-remitting multiple sclerosis, it has shown a protective effect in preclinical studies on stroke (Veltkamp and Gill, 2016). In an early-phase clinical study, oral administration of Fingolimod for three consecutive days following the ischemic event, between 6 and 72 hours after the onset of symptoms, resulted in a significant reduction in lesions and vascular permeability when combined with standard management of the condition (Fu et al., 2014). The efficacy of Fingolimod has been evaluated in a limited number of pilot studies (either alone or in combination with thrombolysis) confirming a beneficial effect on infarct size and neurological functions (Fu et al., 2014; Zhu et al., 2015).

## Conclusion

**Figure 1:**
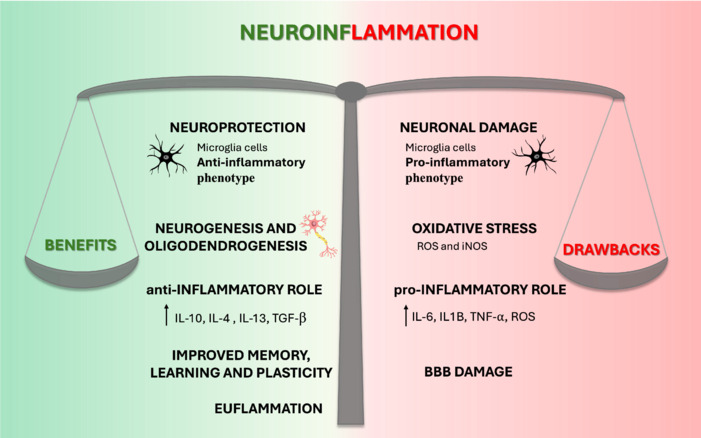
The double-edged sword role of neuroinflammation in ischemic stroke. It represents the dual role of neuroinflammation in ischemic stroke, symbolized by a balanced scale. On the left side, labeled “Benefits,” neuroinflammation is associated with neuroprotection, anti-inflammatory microglial phenotypes, and processes such as neurogenesis, oligodendrogenesis, improved memory, learning, and plasticity, supported by anti-inflammatory cytokines (e.g., IL-10, IL-4, IL-13, and TGF-β). On the right side, labeled “Drawbacks,” it highlights the detrimental aspects, including neuronal damage, oxidative stress (mediated by ROS and iNOS), pro-inflammatory cytokines (e.g., IL-6, IL-1β, TNF-α), and BBB damage. The central scale reflects the delicate balance between these beneficial and harmful outcomes. BBB: Blood–brain barrier; IL: interleukin; iNOS: inducible nitric oxide synthase; ROS: reactive oxygen species; TGF: transforming growth factor; TNF: tumor necrosis factor.

Neuroinflammation exerts a dual role in ischemic stroke (**Figure 1**). Indeed, it plays a crucial role in the pathogenesis of ischemic stroke, impacting both onset and progression. On one hand, it can serve a protective function by aiding in the removal of damaged tissue and restoring cerebral balance; on the other hand, it can also contribute to additional damage by amplifying the injury and promoting neurodegeneration. This dual role makes neuroinflammation a promising therapeutic target. As a result, therapeutic approaches aimed at modulating inflammation in the context of ischemic stroke are attracting increasing interest as effective means to mitigate damage and promote neurological recovery. The dual role of neuroinflammation is evident as discussed in the available immunomodulatory therapeutic strategies, as some of them can mitigate the damage induced by the ischemic event by acting on innate immunity, while others, on the contrary, promote the regeneration and restoration of the brain tissues by influencing the adaptive immune response. A better therapeutic perspective is certainly provided by the latter, as they act on the adaptive immune response, resulting in more effective and sustainable over time. Although some immunomodulators have shown efficacy in animal experiments and clinical studies, most of them have failed in clinical trials despite positive results in experimental studies on ischemic cerebrovascular disease. One possible outcome could result from the adoption of combined therapies, targeting various pathways of ischemic damage, capable of providing greater benefits compared to strategies focused on individual mechanisms of ischemic injury (Chen et al., 2017; Endres et al., 2022).

In conclusion, future investigations should focus on the specific mechanisms of immune-inflammatory responses, assess the appropriate timing of administration, and establish targeted interventions and methodologies. This will help us to better understand the complex role of neuroinflammation in ischemic stroke and to develop more effective therapeutic strategies.

## Data Availability

*Not applicable*.
